# An Intravenous Pharmacokinetic Study of Cannabidiol Solutions in Piglets through the Application of a Validated Ultra-High-Pressure Liquid Chromatography Coupled to Tandem Mass Spectrometry Method for the Simultaneous Quantification of CBD and Its Carboxylated Metabolite in Plasma

**DOI:** 10.3390/pharmaceutics16010140

**Published:** 2024-01-20

**Authors:** Nathan Koch, Olivier Jennotte, Anna Lechanteur, Marine Deville, Corinne Charlier, Jean-Michel Cardot, Patrice Chiap, Brigitte Evrard

**Affiliations:** 1 Laboratory of Pharmaceutical Technology and Biopharmacy, Center for Interdisciplinary Research on Medicines (CIRM), University of Liège, 4000 Liège, Belgium; ojennotte@uliege.be (O.J.); anna.lechanteur@uliege.be (A.L.); b.evrard@uliege.be (B.E.); 2 Department of Toxicology, Center for Interdisciplinary Research on Medicines (CIRM), Academic Hospital of Liège, 4000 Liège, Belgium; m.deville@chuliege.be (M.D.); c.charlier@chuliege.be (C.C.); patrice.chiap@chuliege.be (P.C.); 3 Borvo, 63211 Ceyrat, France; jean-michel.cardot@wanadoo.fr

**Keywords:** cannabidiol, intravenous pharmacokinetic, carboxy-cannabidiol, cyclodextrin, piglet plasma, UHPLC–MS/MS, validation

## Abstract

Cannabidiol (CBD) has multiple therapeutic benefits that need to be maximized by optimizing its bioavailability. Numerous formulations are therefore being developed and their pharmacokinetics need to be studied, requiring analytical methods and data from intravenous administration. As CBD is susceptible to hepatic metabolism, the requirement of any method is to quantify metabolites such as 7-COOH-CBD. We demonstrated that CBD and 7-COOH-CBD could be simultaneously and correctly quantified in piglet plasma by using an UHPLC–MS/MS technique. The validated method allowed for an accurate bioanalysis of an intravenously injected solution consisting of CBD-HPβCD complexes. The experimental pharmacokinetic profile of CBD showed multi-exponential decay characterized by a fast apparent distribution half-life (0.25 h) and an elimination half-life of two hours. The profile of 7-COOH-CBD was not linked with the first-pass metabolism, since 80% of the maximum metabolite concentration was reached at the first sampling time point, without any decrease during the period of study. A two-compartment model was optimal to describe the experimental CBD profile. This model allowed us to calculate macro–micro constants and volumes of distribution (V_ss_ = 3260.35 ± 2286.66 mL) and clearance (1514.5 ± 261.16 mL·h^−1^), showing that CBD is rapidly distributed to peripheral tissues once injected and slowly released into the bloodstream.

## 1. Introduction

Cannabidiol (CBD) is a promising natural compound extracted from *Cannabis sativa* that shows significant pharmacological properties [1]. CBD has limited activity at cannabinoid receptors CB1 and CB2, and its therapeutic effects are rather linked to interactions with other receptors such as transient receptor potential vanilloid (TRPV) channels, serotonin (5-HT_1A_) receptors or adenosine A2A receptors [2,3]. As an example, CBD limits inflammatory responses by increasing adenosine, and its role in TRPV1 has an impact on neuroprotection, inflammation and anti-convulsing effects. Although authors have explored its potential to treat various conditions as diverse as schizophrenia, anxiety, sleep disorder and pain [4], only two drugs are currently on the market and are used to treat rare diseases such as epilepsy-related Lennox–Gastaut or Dravet syndrome. Depending on the study and the clinical target, the proposed posology varies from a few milligrams per kilogram to several hundred milligrams. In general, the ideal dose is individually adjusted during treatment to achieve the clinical goal [5,6]. The pharmacokinetics of CBD have been reviewed in humans, showing that AUC_0-t_ and C_max_ are dose-dependent, on the contrary to the T_max_, which occurs between 0 and 5 h depending on the administration route [7]. The half-life of elimination (t_1/2_) is generally around one or two hours for pulmonary or single oral administration, and may reach several days after chronic oral administration. Clearance values are also reported to be administration-dependent, for example, 74.4 L·h^−1^ following an intravenous injection compared to a range of 2546 to 4741 L·h^−1^ in a fasted state following oromucosal spray administration. This review also highlights the paucity of intravenous data, with only one study described in this work. Several pharmacokinetic studies of orally administered CBD have been performed in other species such as canines [8], rats [9] and horses [10], and only a few have investigated the intravenous route. For example, Xu et al. intravenously injected a CBD emulsion in mice at a dose of 10 mg/kg and showed a half-life of 3.9 h and a clearance of 3.4 L·h^−1^·kg^−1^ [11]. Turner et al. administered intravenous CBD dissolved in DMSO at a dose of 0.1 mg/kg to horses and showed a half-life of 3.15 h and a volume of distribution at steady state of 5481.7 L/kg [12]. CBD is a highly lipophilic drug (LogP = 6.3) and suffers from a high first-pass metabolism and a poor aqueous solubility, resulting in a low and erratic bioavailability [13,14]. Research is therefore being conducted to develop CBD formulations that improve its aqueous solubility, stability, absorption and, thus, ultimate bioavailability [15,16,17,18]. As a BCS (biopharmaceutics classification system) II molecule, CBD has been formulated with excipients such as hydrophilic polymers, cyclodextrins, mesoporous silica or lipid excipients, and successfully demonstrated their benefits under in vitro conditions [16,19,20]. However, these encouraging works have to be confirmed under in vivo conditions. While much work is being carried out to minimize the use of animals, no in vitro model has yet been fully developed to study the liver first-pass metabolism, and it is difficult to overcome in in vivo models to demonstrate the increased bioavailability. Complete pharmacokinetic data such as C_max_, T_max_, the volume of distribution or the exploration of the entero-hepatic cycle generally require animal studies [21]. In vivo studies can be performed in humans as well as in rats, rabbits, dogs, guinea pigs or even piglets. The latter species is valued for its large blood volume, which allows for extended sampling, and is relatively similar to humans in terms of characteristics such as cytochrome P450 (CYP) metabolism enzyme patterns and digestive function [22]. In order to precisely characterize the absorption in per os studies, as well as the real distribution and the real elimination of a drug, intravenous pharmacokinetics is required to dispose of data based on full bioavailability without any interference from the absorption process.

**Figure 1:**
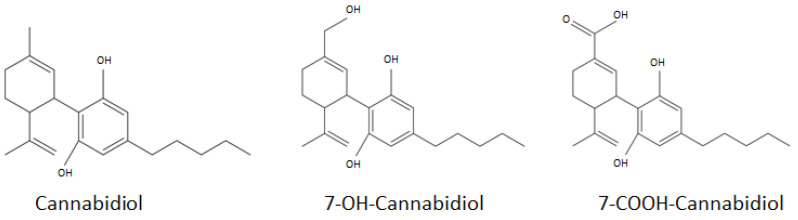
Chemical structure of cannabidiol, 7-OH-cannabidiol and 7-COOH-cannabidiol.

Since the bioavailability of CBD can be increased by strategies that overcome or limit its first-pass metabolism, it is interesting to quantify some of the metabolites. CBD is indeed highly metabolized in the liver to the more hydrophilic and active compound 7-OH-cannabidiol, which in turn is oxidized to the inactive compound 7-COOH-CBD, as shown in Figure 1 [23].

These oxidations, mainly performed by CYP2C19 and CYP3A4, lead to glucuronidation, which results in fecal and urinary excretion [24]. Few authors have followed the evolution of CBD and 7-COOH-CBD during pharmacokinetic studies. For example, Devinsky et al. developed a CBD formulation for inhalation and compared its pharmacokinetic properties with an oily CBD solution. They showed that the inhaled formulation had an increased AUC_last_ with very little formation of 7-COOH-CBD, in contrast to the oily solution [23]. To the best of our knowledge, there is no published validated method for the simultaneous quantification of CBD and one of its major metabolites, 7-COOH-CBD, in piglet plasma, while validated methods exist for the quantification of the concerned compounds in human plasma [25,26,27]. Due to the low bioavailability of cannabinoids, many biological cannabinoid concentrations are typically on the nanoscale (ng/mL). In addition, cannabinoids regroup many similar compounds that can be difficult to identify and separate. Ultra-high-pressure liquid chromatography coupled to tandem mass spectrometry (UHPLC-MS/MS) is a powerful tool to address these issues, as it allows for high sensitivity, high resolution and high selectivity [28]. Therefore, the aim of the present work is to determine, for the first time, the intravenous pharmacokinetic parameters of CBD in piglets through the application of a UHPLC-MS/MS method for the simultaneous quantification of CBD and 7-COOH-CBD. The experimental pharmacokinetic profiles of CBD and 7–COOH–CBD are first discussed. Then, the optimal pharmacokinetic compartmental model of CBD is discussed and the metabolization, distribution and clearance of CBD are investigated by using the chosen compartmental model.

## 2. Materials and Methods

### 2.1. Chemicals

Cannabidiol powder was purchased from THC Pharm GmHB (Frankfurt am Main, Germany). Cannabidiol-d_3_, 7-COOH-cannabidiol and 7-COOH-cannabidiol-d_3_ methanol solutions were purchased from LGC Ltd. (Teddington, UK) and Cerilliant Corporation (Round Rock, TX, USA), and stored at −20 °C. Piglet plasma was collected and purchased from PigforLife (Marloie, Belgium). Methanol and water were purchased from J.T. Baker by Filter Service (Eupen, Belgium), ethyl acetate was purchased from VWR Chemicals (Leuven, Belgium) and n-hexane 95% was purchased from Macron Fine Chemicals (VWR, Leuven, Belgium). All solvents were LCMS-grade. Sodium hydroxide and glacial anhydrous acetic acid 100% were purchased from Merck (Darmstadt, Germany); ammonium bicarbonate and ammonia 25% were obtained from Sigma-Aldrich (Saint Louis, MO, USA) and Fisher Chemicals (Hampton, NH, USA), respectively. HP-β-CD (Kleptose^®^, substitution degree of 0.62; Mw 1470 g/mol) was purchased from Roquette (Lestrem, France).

### 2.2. Instrumentation and Method

The mass spectrometer was a Water Xevo^TM^ TQ-S mass spectrometer equipped with an electrospray ionization source (ESI) operating in the positive ion mode and interfaced with a Waters Acquity UPLC I-Class inlet system. Data acquisition was achieved using the MassLynx Version 4.2 software and the TargetLynx version 4.2 software. The chromatographic conditions were set with an Acquity^®^ UPLC BEH C18 (1.7 µm (2.1 × 50 mm)) column associated with a guard filter as the stationary phase, and the mobile phase consisted of a mixture of an ammonium bicarbonate 10 mM buffer (pH 10) and methanol in a gradient mode as shown in Table 1. The temperature was set at 45 °C for the column and 10 °C for the autosampler. The flow rate was set at 0.45 mL/min and the injection volume was 1 µL. The run time was set at 6 min.

Detection was carried out with tandem mass spectrometry using a Xevo-TQS. The positive electrospray ionization mode was used (+1 kV) with a desolvation gas flow of 1000 L/h (nitrogen) and a collision gas flow of 0.20 mL/min (argon). The temperature of the source was set at 120 °C, while the desolvation temperature was set at 500 °C. The analytes were identified and quantified using the multiple reaction monitoring (MRM) mode. Two MRM transitions (*m*/*z*) were used for the identification and quantification of CBD and 7-COOH-CBD. The cone voltage, collision energy (eV) and retention time (RT) of each compound are detailed in Table 2.

### 2.3. Preparation of Standard Solutions

Standards solutions were prepared in methanol at four different concentration levels—SF1 (500 ng/mL of CBD and 5000 ng/mL of 7-COOH-CBD), SF2 (250 ng/mL of CBD and 2500 ng/mL of 7-COOH-CBD), SF3 (50 ng/mL of CBD and 500 ng/mL of 7-COOH-CBD) and SF4 (5 ng/mL of CBD and 50 ng/mL of 7-COOH-CBD)—as well as an internal standard solution (SI; 250 ng/mL of CBD-d_3_ and 2000 ng/mL of 7-COOH-CBD-d_3_), which were stored at −20 °C.

### 2.4. Calibration, Validation and QC Samples

The calibration standards, validation standards (5 × 4 concentration levels) and quality control samples were prepared in glass tubes with a conical bottom, as shown in the Supplementary Materials (Tables S1–S3).

### 2.5. Sample Preparation

We prepared 10% acetic acid (*v*/*v*) daily by dissolving 10 mL of 100% anhydrous glacial acetic acid with 100 mL of ultra-pure water. Hexane/ethyl acetate (9/1 *v*/*v*) and methanol/water (50/50 *v*/*v*) were prepared and used for a maximum of one week.

A total of 500 µL of plasma (calibration, validation, quality control or pharmacokinetic samples) was fortified with 20 µL of SI, 100 µL of 10% acetic acid and 5 mL of hexane/ethyl acetate (9/1 *v*/*v*). The samples were stirred for 10 min with a tube stirrer and then centrifuged for 10 min at 2000 rpm. The supernatant was transferred to a glass vial prior to evaporation in nitrogen at 40 °C. Once dried, 100 µL of methanol/water (50/50 *v*/*v*) was added to the glass vial to dissolve the residue; transferred to Eppendorf centrifugation tubes and centrifuged at 18,000 rpm for 10 min; and finally transferred into UHPLC vials.

### 2.6. Method Validation

The method’s validation was based on ICH guideline M10 for bioanalytical method validation. This includes, notably, the following elements: selectivity, specificity, matrix effect, calibration curve, lower and upper limits of quantification (LLOQ and ULOQ), accuracy, precision, carry-over, dilution integrity and stability. These validation elements, along with their corresponding specifications, can also be found in the literature [29,30,31].

Selectivity was assessed by analyzing piglet plasma from five different animals. Guidelines recommend using at least six individual sources, but the use of fewer may be acceptable in the case of rare matrices. In our case, only five animals were available. No interfering peak should be detected with a surface area superior to 20% of the lower limit of quantification (LLOQ) and 5% of the internal standard. Specificity, which is the ability of the method to detect and differentiate the analytes from other related substances, was determined through the chromatographic separation and detection of the analytes and their deuterated related compounds. Specificity was assessed by determining the coefficients of variation for the ion ratios of CBD and 7-COOH-CBD by considering the transitions for quantification and identification.

For the test item and the internal standard, the matrix factor (MF) was calculated for each lot of matrices by determining the ratio of the peak area in the presence of the matrix (measured by analyzing a blank matrix spiked after extraction with analytes) to the peak area in the absence of the matrix (pure solution of analytes). This determination was made at low and high levels of concentration. The internal standard (IS) normalized MF was also calculated by dividing the MF of the test item by the MF of the IS. For each individual matrix source, accuracy should be within ±15% of the nominal concentration and the precision should not be greater than 15%. Carry-over was studied by analyzing blank samples after injecting a calibration standard at the ULOQ.

Accuracy and precision were also determined through the data obtained with three series on independent days. The acceptance limits of the accuracy profiles were set at ±20% of the nominal concentrations, and the tolerance limit was set at 17.5% in accordance with the literature [32,33]. The tolerance limit was evaluated through the percentage of risk of having measurements fall outside of the acceptance limits for each level of concentration. The precision was expressed through the repeatability and intermediate precision. The correspondent relative standard deviation (RSD%) should not be greater than 15%. The LLOQ and ULOQ were determined through the calibration curve, obtained on three independent runs over three days.

The dilution integrity was evaluated by preparing five independent solutions of 100 ng/mL and 1000 ng/mL of CBD and 7-COOH-CBD, respectively, by diluting 100 µL of SF1 with 400 µL of blank plasma. To be in accordance with the practical dilution, 100 µL of the prepared solution was diluted with 400 µL of blank plasma with a concentration target of 20 ng/mL for CBD and 200 ng/mL for 7-COOH-CBD. Integrity was assessed if results were within ±15% of the concentration target.

Finally, freeze–thaw, bench-top, autosampler and long-term stability were assessed. Low and high QC concentrations in piglet plasma were used to demonstrate the freeze–thaw stability (at least 12 h between each cycle) at −80 °C, as well as autosampler stability (12 h, 10 °C), bench stability (4 h, 20 °C) and long-term stability (8 months, −80 °C).

### 2.7. Data Treatment

Excel results were treated and submitted to E-noval^TM^ V3.0 (Pharmalex, Mont-Saint-Guibert, Belgium) for the choice of the calibration model and the method validation.

### 2.8. Application of the Method

#### 2.8.1. In Vivo Study

The in vivo study was performed within the PigForLife^®^ (CER Group Facilities) GLP structure (Marloie, Belgium). The study protocols were approved by the Local Ethical Commission before starting the study and complied with the Belgian regulation as published in the «Royal Decree of 29 May 2013 on the protection of experimental animals, A.G.W. 30 November 2017».

Sterile and isotonic CBD:HP-β-CD 100 mM (1.2 mg CBD/mL) solutions were prepared. A HP-β-CD stock solution (200 mM) was prepared by dissolving 31.0 g of HP-β-CD up to 100 mL with milliQ water. CBD:HP-β-CD complexes were prepared by adding an excess of CBD (45 mg) powder to 5 mL of milliQ water and 5 mL of the HP-β-CD stock solution, added with 84 mg of NaCl. The suspensions were kept in a rotating water bath set at 37 °C for 12 h. The suspensions were finally filtered through a 0.2 µm PHENEX^®^ PTFE filter in laminar flow insulator conditions. The osmolarity and the pH value of the solutions were determined using a Freezing Point Osmometer (Löser, Delta Labo, Avignon, France) and a pH meter (Seven Easy, Mettler Toledo, Zaventem, Belgium), respectively. The solutions were injected to three Large White piglets (male and female, between 8 and 10 kg) at a dose of 0.3 mg/kg, as detailed in Table 3.

The animals were born on site and used in good health. The animals were housed together in pens of about 40 m^2^ and an enriched environment was provided. Natural temperature and light cycles were used in a straw litter. During storage, the animals were fed ad libitum with a balanced pellet diet and were not given any curative or prophylactic veterinary drugs or additives during the study period. Tap water was available ad libitum. The piglets were fasted overnight (food but no water) before receiving the formulations. All piglets were clinically examined before the study and before sampling. Blood samples (2 mL in EDTA tubes) were taken at 5 min, 10 min, 20 min, 30 min 45 min, 60 min, 90 min, 2 h, 4 h and 8 h. Blood samples were stored at 4 °C and centrifuged at 2500× *g* for 10 min. Plasma samples were then frozen at −80 °C until their analysis.

#### 2.8.2. In Vivo Data Treatment

Pharmacokinetic analyses were evaluated by using the software Phoenix WinNonlin 8.4 version (Certara, Princeton, NJ, USA). C_max_ and AUC_last_ were determined through a non-compartmental pharmacokinetic analysis using the linear trapezoidal linear interpolation calculation method. Obtained values were normalized do the dose administered. The volumes of distribution (V_1_ and V_ss_), clearance and macro (A, B, C, α, β, γ) and micro (k_10_, k_12_, k_21_, k_13_, k_31_) constants were determined assuming a 2- or 3-compartment model. The model offering the best compromise between fitting and the number of parameters was selected based on an F test approach.

## 3. Results and Discussion

This method was adapted from a validated and routinely used method for the quantification of CBD, Δ-9-tetrahydrocannabinol and some of their related metabolites in human saliva, urine, blood and plasma matrices in a laboratory of toxicology (Academic Hospital of Liège, Department of Toxicology, Liège, Belgium). This work aimed to transfer this knowledge to simultaneously quantify CBD and an important metabolite, 7-COOH-cannabidiol, in piglet plasma samples. As the sample preparation involves a liquid–liquid extraction (LLE) process, 10% acetic acid was added to the samples to ensure the nonionic form of CBD, and in particular, 7-COOH-CBD. In fact, LLE requires unionized analytes to be suitable [31]. 

**Figure 2:**
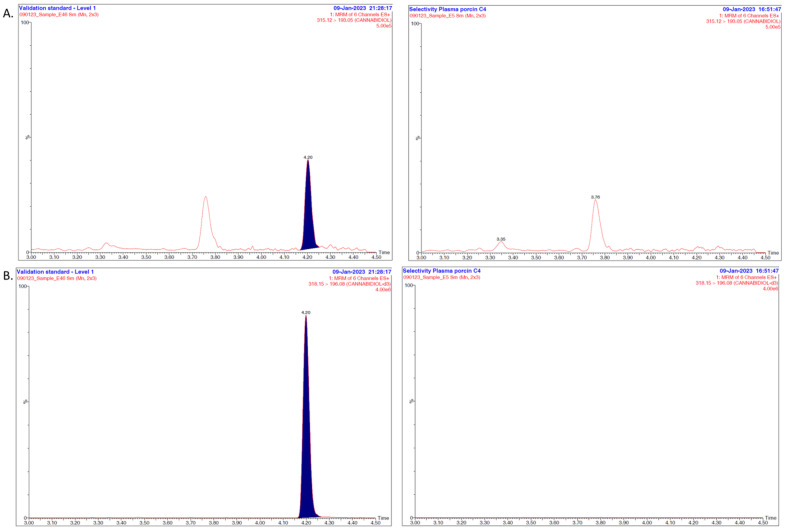

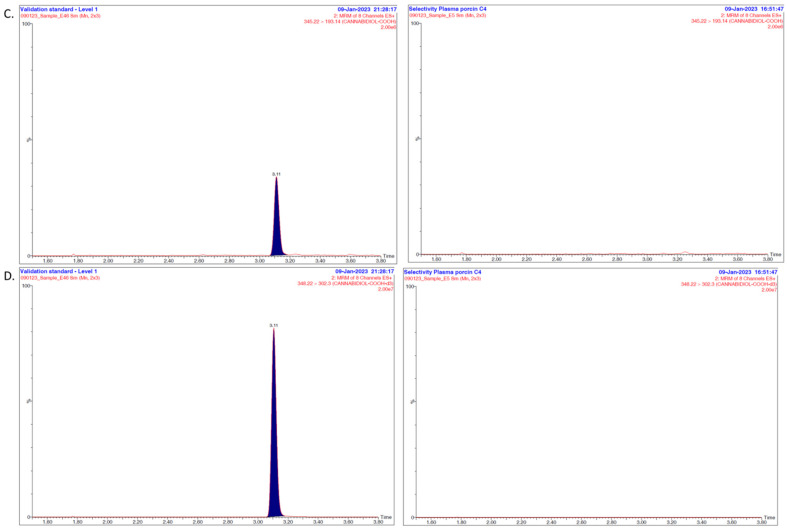
(**A**) Determination of the method’s selectivity: example of MRM chromatograms for cannabidiol obtained after analysis of a piglet plasma sample spiked at the LLOQ level (**left**) and a blank plasma sample (**right**). (**B**) Determination of the method’s selectivity: example of MRM chromatograms for cannabidiol-d_3_ obtained after analysis of a piglet plasma sample spiked at the LLOQ level (**left**) and a blank plasma sample (**right**). (**C**) Determination of the method’s selectivity: example of MRM chromatograms for 7-COOH-CBD obtained after analysis of a piglet plasma sample spiked at the LLOQ level (**left**) and a blank plasma sample (**right**). (**D**) Determination of the method’s selectivity: example of MRM chromatograms for 7-COOH-CBD-d_3_ obtained after analysis of a piglet plasma sample spiked at the LLOQ level (**left**) and a blank plasma sample (**right**).

The selectivity of the method was assessed based on the absence of any interfering endogenous components of piglet plasma. The plasma matrices were spiked with the analytes to reach the first concentration level of the validation standards, which actually corresponds to the LLOQ for both CBD and 7-COOH-CBD. For each analyte, measured with the concerned transition, no interfering peak was detected, demonstrating that the method can discriminate the peaks of interest from all piglet plasma-related substances, as shown in the obtained chromatograms (Figure 2A–D).

Concerning the specificity, the coefficient of variation for the ion ratio was 6.0% and 14.6% for CBD and 7-COOH-CBD, respectively, therefore showing the appropriate separation and detection of the analytes.

The matrix effect is the possibility of the co-elution of compounds related to the matrix that can interfere with the electrospray ionization process. It can inhibit or enhance the ionization process and influence the targeted signal, and thus cause failure. The IS normalized matrix effects concerning CBD were 0.89 ± 0.09 (CV% = 10.2) and 1.10 ± 0.08 (CV% = 7.4) for low and high QC, respectively. For 7-COOH-CBD, values of 0.89 ± 0.09 (CV% = 9.7) and 1.09 ± 0.08 (CV% = 7.4) were obtained for low and high QC, respectively, suggesting that the plasma matrix of the piglets does not significantly interfere with the detection process. The carry-over test did not show any suspicious peak in the blank samples despite the previous injection of a high concentration of CBD and its metabolite.

Different regression models were fitted, and the weighted ($\frac{1}{x^{2}}$) linear regression model was selected for both compounds, as it is the simplest model giving the lowest relative errors at each concentration level of the validated range. The linearity of the method for both CBD and 7-COOH-CBD is shown in the Supplementary Materials (Table S4). The determination coefficient value (r^2^) is > 0.99 for both compounds, demonstrating the adequate linearity in the desired dosing range, 0.5–50 ng/mL for CBD and 5–500 ng/mL for 7-COOH-CBD.

**Figure 3:**
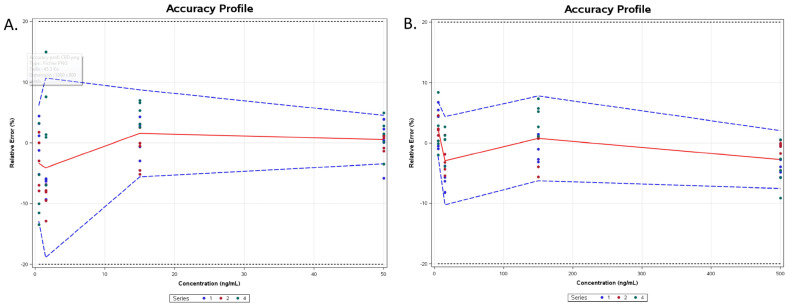
Accuracy profile for CBD (**A**) and 7-COOH-CBD (**B**).

For method accuracy, the concept of total error (including both bias and standard deviation for intermediate precision) was considered. It was assessed from an accuracy profile (Figure 3), which was obtained by linking, on the one hand, the lower bounds and, on the other hand, the upper bounds of the β-expectation tolerance limits calculated at each concentration level of the validation samples. The acceptance limits with respect to the total error of measurement were set at ±20%. The method was considered as being valid within the range, because the accuracy profile was within the acceptance limits irrespective of the concentration level. As can be seen in Figure 2, all values were within ±15% except at the LLOQ level for CBD. It is in accordance with FDA guidelines [30]. Moreover, the risk of having measurements fall outside of the acceptance limits was below 17.5% for both CBD and 7-COOH-CBD at each tested level of concentration, proving that the method is therefore accurate over the concentration range.

The relative bias (%) from five replicates of validation standards provided by the same series (intra-day) and validation standards provided from different series (inter-day) are shown in Table 4. No value exceeds 15%, suggesting satisfying criteria. The method’s trueness is confirmed.

The method’s precision was assessed with the relative standard deviation (RSD%) between five replicates of validation standards from the same series (intra-day), known as repeatability, and with the relative standard deviation (RSD%) of time-different validation standards provided from different series (inter-day). The repeatability (RSD%) and intermediate precision (RSD%) presented in Table 5 prove the good intra/inter-day precision. The highest RSD value was obtained with CBD at a low concentration (1.5 ng/mL), but still acceptable, since it is far from 15%.

Since some samples of plasma were expected to exceed the ULOQ, a dilution process had to be added to the treatment of some samples. The dilution integrity was measured and showed adequate results, as the targeted concentration was successfully measured (Table 6). A five-fold dilution is therefore adequate to accurately measure CBD and its carboxylated metabolite, allowing samples to be quantified at maximum concentrations of 250 ng/mL for CBD and 2500 ng/mL for 7-COOH-CBD.

The stability of CBD and its metabolite within the plasma matrix during storage was investigated to ensure the quality of the obtained results. Indeed, samples are generally not directly analyzed once collected and must not degrade upon their storage conditions. In order to anticipate any technical problems during analysis that might block samples at different temperatures (such as those of the autosampler), it is useful to show that the practical use and treatment of samples at room temperature does not influence the results. All samples assayed under the different conditions were within ±15% of the target concentrations. Piglet plasma samples can therefore be stored for at least 8 months at −80 °C and can be handled safely and conveniently if they are manipulated at room temperature for no more than four hours. Samples can also be correctly reanalyzed after thawing and refreezing.

### 3.1. Overall Discussion of the Bioanalytical Method

The present method offers the possibility to quantify CBD and its secondary metabolite, 7-COOH-CBD, in piglet plasma rapidly and with high accuracy and precision. The metabolite plasmatic concentration is reported in the literature to be 40-fold higher than that of CBD [23]. Considering the LLOQ of CBD (0.5 ng/mL), it was decided to increase the dosing range of the metabolite by a factor 10 (LLOQ of 7-COOH-CBD of 5 ng/mL) in order to avoid unnecessary sample dilution. To the best of our knowledge, only a few authors have considered the UHPLC–MS/MS method to adequately quantify CBD and its metabolite, 7-COOH-CBD, and none have worked with piglet plasma [25,26,27,34]. The very low injection volume (1 µL) and the rapid elution of the compounds (3.10 min for 7-COOH-CBD and 4.20 min for CBD) allow an optimal peak shape of both analytes in a very short lapse of time to be obtained. This study also benefits from the use of 7-COOH-CBD-deuterated as an internal standard to quantify the carboxylated metabolite. Indeed, an optimal IS must have similar chemical and physical properties to the studied compound. Due to the unavailability of some deuterated analogs of CBD metabolites, many validations have been carried out by using other cannabinoids as internal standards, such as THC-COOH-d_3_ or THC-OH-d_3_, that show retention times that are relatively far from that of 7-COOH-CBD. The use of the related deuterated compound allows for a reliable quantification of this compound.

### 3.2. Intravenous Study

**Figure 4:**
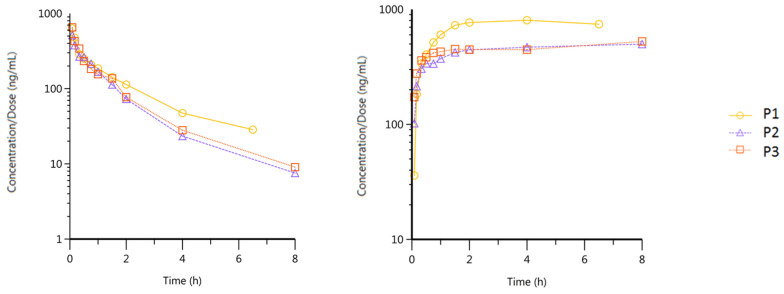
Intravenous pharmacokinetic profile of a CBD-HP-β-CD solution, CBD profile (**left**) and 7–COOH–CBD (**right**) (*n* = 3); please note that the scales are different for the two graphs.

The pharmacokinetic information on intravenously injected formulations is generally highly needed, as it is the only way to obtain values such as distribution and elimination constants or the real terminal half-life elimination time, as well as volumes of distribution and clearance, without any interference from the absorption process. Intravenous data also provide important information on the distribution of the drug in the body. Because CBD is a BCS II molecule, it is challenging to formulate a parenteral solution. A few authors have studied the intravenous kinetics of CBD by injecting Cremophor^®^ emulsion, suspensions containing Tween^®^, or propylene–ethanol solutions in mice or humans [35,36,37]. In our study, the cyclodextrin (CD) strategy was chosen in accordance with previous work in our laboratory [16]. Hydroxypropyl-β-cyclodextrin (HP-β-CD) was used to form a soluble CBD-CD complex, and the optimal formulation contained 1.2 mg of CBD/mL (100 mM of HP-β-CD). To meet physiological conditions, NaCl was added to finally reach 320 mOsm/kg and a pH of 7.4. The preliminary indication of the in vitro drug release tests showed a complete CBD release within 1 min (Supplementary Materials, Figure S1). HP-β-CD was also selected as a cyclodextrin since it has been widely studied and has proven its ability to rapidly and completely release the guest molecule once intravenously injected [38,39]. Once injected, CBD dissociates rapidly and completely from the CD, which is explained by the low stability constant. The CBD:HP-β-CD inclusion complex has already been studied, and an A_L_ type with a stability constant of 146.7 M^−1^ was determined [40]. This low affinity of CBD for this CD allows for a rapid release of CBD through dilution with the bloodstream. Piel et al. compared the intravenous pharmacokinetic profile of miconazole in sheep from a CD solution and a micellar solution and showed that HP-β-CD did not interfere with the release of miconazole and could be proposed as a safe solubilizing agent for parenteral administration [41]. In order to respect the welfare of the animals as much as possible, and taking into account the maximum volume that could be collected, the sampling was maximized at the very beginning of the study to better characterize the possible different slopes and, thus, elimination constants. As this work is an exploratory study of intravenous administrations of CBD in order to estimate the pharmacokinetic model, it was decided to include three piglets due to ethical reasons. One piglet (P1) had to be euthanized before the end of the study due to respiratory distress, resulting in a missing value for the 8 h time point, but an additional time point at 6.5 h (Figure 4).

The role of CBD or its metabolites in this premature death may be questionable. This cannabinoid is currently being studied in various pulmonary pathologies and shows positive effects. For example, the authors demonstrated an improvement in lung structure and anti-inflammatory effects in the context of acute respiratory distress syndrome (ARDS) [42]. Stress caused by handling is the most likely cause of this unfortunate death.

In addition, the aforementioned piglet presented less consistent behavior in comparison with the two other animals (P2 and P3). A slower elimination rate combined with a faster apparition of metabolites was noticed. P1 showed signs of failure that could alter its blood flow rate and renal and hepatic functions. These alterations could explain the difference from P2 and P3, since their distribution and metabolization pathways may be different. Another difference is that the CBD solution had to be injected into P1 via the cranial vena cava as it was not possible to find a suitable auricular vein. The CBD profile exhibits multi-exponential decay with a fast apparent distribution, translated as a distribution half-life of 0.25 h and an elimination half-life of around two hours. A C_max_ of 599.83 ± 75.85 ng/mL and an AUC_last_ of 639.02 ± 79.22 ng·h·mL^−1^ were calculated. The simultaneous quantification of the metabolite 7-COOH-CBD showed an accumulation without any decrease within the duration of the study, while appearing very quickly. Indeed, the formation of the metabolite was surprisingly faster than expected and not linked with the first-pass metabolism. Metabolite concentrations were observed as early as 5 min, and 80% of the maximum measured value was observed between 0.75 and 1.5 h for all pigs. No apparent terminal elimination of 7-COOH-CBD could be calculated since the concentration at the last sampling was the C_max_ (609.79 ± 169.19 ng/mL). The calculated AUC_last_ of the metabolite was 3926.68 ± 571.99 ng·h·mL^−1^. As a secondary metabolite, it also depends on the kinetic formation of 7-OH-CBD. Interestingly, and thanks to the present validated quantification method, this study shows that CBD is highly and rapidly metabolized even in the absence of first-pass metabolism. After IV administration, 7-COOH-CBD is present from the first sampling point, i.e., 5 min. CBD must be distributed almost instantaneously to the tissue that activates its metabolism. The present results therefore highlight the high and rapid systemic metabolization of CBD.

The experimental pharmacokinetic profile of CBD was then modeled. Due to the non-linearity of the log scale graph, mono-compartmental behavior is not expected. This seems logical due to the high lipophilicity of CBD, which favors the distribution of this cannabinoid in many tissues, especially adipose tissues. Fitting models with two and three compartments were therefore tested with a weighting of 1/y^2^ to better estimate the lowest values. In addition, the comparison of the use or not of piglet 1 was performed (Table 7).

The terminal phase (B/β for a two-compartment model and C/γ for a three-compartment model) represents the apparent elimination of the drug, excluding absorption (if not via the intravenous route) and distribution. The choice of the model to be used and the number of animals may be discussed. There is no significant difference in the macro constants calculated with two or three animals (unpaired *t*-test, *p*-values > 0.5). The elimination values of CBD are not influenced by the number of used animals despite the non-consistent behavior of P1 compared to P2 and P3. The three-compartment models give better Akaike Information Criterion (AIC) values and R^2^ values than the two-compartment models. This is quite logical, since adding parameters improves the fit. However, it is interesting to consider the benefits of such a model with three macro constants. Since the aim of these values is to describe the distribution and elimination of CBD, but also its possibility to be used in pharmacokinetic studies, the complexity of using more than two compartments must be justified. As a reminder, compartment modeling is a hypothetical way to describe and interpret experimental data. A multi-compartmental (two or more) model indicates a high distribution of the drug in poorly vascularized tissue such the brain, fat or muscle, contrarily to mono-compartmental modeling. However, real living bodies counter multimillions of compartments, which are, of course, impossible to model, and no multi-compartment model can describe and identify the real distribution [43]. The benefit of using three-compartment models can be estimated by calculating the Fisher test value (1) (Table 8).

**Figure 5:**
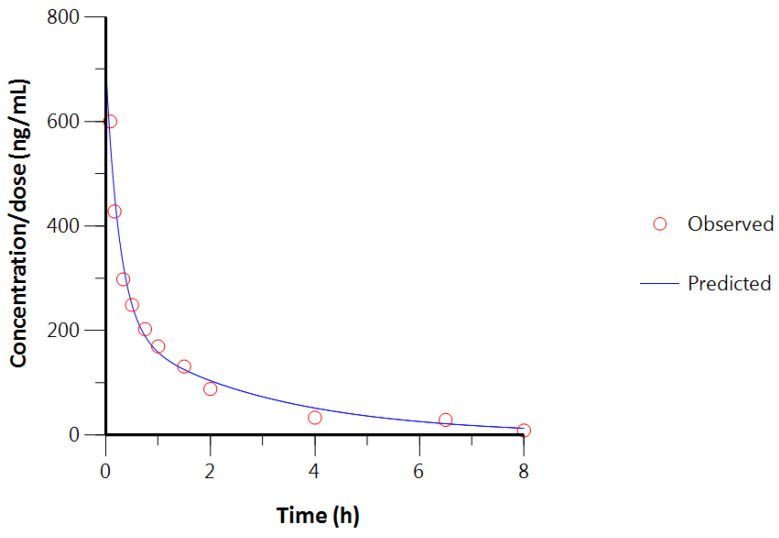
Predicted and observed (*n* = 3) CBD concentrations after IV injection.

Since the F value is not larger than F_Table for α = 0.05_, the three-compartment model is not statistically superior to the two-compartment model. In order to facilitate the analyses while optimally describing the in vivo fate of CBD once it has been injected, the two-compartment model is the best model to use. Figure 5 shows the predicted values when using the concerned model compared to the observed values. It is clear that the prematurely dead piglet increases the variability of the values. However, it also represents the variability in real life that occurs due to inter- and intra-variability and has to be taken into account.

The different volumes of distribution, micro-constants and clearance were evaluated by using values analyzed with a two-compartment model (Table 9).

The capacity and depth of the peripheral distribution were estimated by using the micro constants. The depth value of 0.58 means that CBD is more rapidly distributed in the peripheral tissues than it is eliminated from the body. The capacity value of 1.5 translates that CBD tends to leave a tissue more slowly than its arrival, which is rapid. After a single administration, this cannabinoid is thus rapidly diffused to non-central compartments but is relatively slowly retransferred again to plasma compartments and cleared. This is also demonstrated by comparing the volume of distribution at steady state (3260.35 ± 286.66 mL), representing the total volume, with the central distribution volume (1512.43 ± 538.41 mL). Since the V_ss_ is more than twice the V_1_ value, it can be concluded that CBD is highly diffused in non-central compartments. Considering a blood volume of approximately 60 mL/kg in the piglet species [44] and the mean weight of the piglets used in this study (9.11 kg), V_1_ also shows a high CBD distribution in tissues that are well perfused. To estimate the ratio of the drug’s quantity in the central compartment over the drug’s quantity in the body (*f_c_*), Equation (2) shows the relation between β (the elimination process description) and *k*_1-0_ (the rate constant of elimination):

(2) $$f_{c}=\frac{\beta }{k_{1\text{-}0}}$$

The calculated value for CBD is 0.33 and indicates that two thirds of the quantity of CBD is distributed outside the plasma compartment.

Peripheral tissues act as tanks which are rapidly charged and progressively release CBD into the bloodstream after IV injection. The clearance value (1514.5 ± 261.16 mL·h^−1^) in piglets could be determined thanks to this intravenous study. The relatively low value can be analyzed in parallel with the rapid formation of 7-COOH-CBD, highlighted by the present validated method. While the appearance of the 7-COOH-CBD metabolite is very rapid, it should not be forgotten that it is a secondary metabolite. The formation of the primary 7-OH-CBD metabolite is even more rapid. It indicates that CBD is rapidly transformed and cleared once in plasma. The clearance values therefore support the idea that CBD is strongly bound to peripheral tissues and protected in some way from systemic metabolism during the time it resides in these tissues, without which the clearance value would be much higher.

## 4. Conclusions

The method presented has been successfully validated and may be very useful for further in vivo studies conducted in piglet plasma. It allows for an accurate and simultaneous quantification of CBD and its major secondary metabolite, 7-COOH-CBD, which requires a very small injection volume and is rapid, with retention times of approximately 3 and 4 min. The applicability of the method was demonstrated by determining, for the first time, the intravenous pharmacokinetic parameters of CBD:HP-β-cyclodextrin solutions in piglets. The simultaneous monitoring of CBD metabolites, obtained directly with the CBD’s quantification, showed a very fast drug metabolization while avoiding the first-pass effect. The accurate quantification of CBD allows the a discussion and selection of the optimal compartment model to use, and the distribution of CBD within the body has been described. Finally, this study provides important information on CBD elimination rates that are necessary for many applications.

## Figures and Tables

**Table 1:** Gradient mode.

Time (min)	Mobile Phase A (Ammonium Buffer, pH 10)	Mobile Phase B (Methanol)	Mode
0.0–1.0	70%	30%	Isocratic
1.0–4.5	3%	97%	Gradient
4.5–5.0	3%	97%	Isocratic
5.0–5.05	70%	30%	Gradient

**Table 2:** Mass spectrometry parameters.

Analytes	Precursor Ion (*m*/*z*)	Product Ion (*m*/*z*)	Cone Voltage (V)	CE (eV)	RT (min)
CBD	315.1	193.1 (Quantification) 259.1 (Identification)	25 25	20 20	4.20
CBD-d_3_	318.2	196.1	20	20	4.19
7-COOH-CBD	345.2	193.1 (Quantification) 299.2 (Identification)	40 40	28 16	3.10
7-COOH-CBD-d_3_	348.2	302.3	34	24	3.09

**Table 3:** In vivo injection protocol.

Piglet	Weight (kg)	Volume Injected (mL)	Injection Route
P1	9.45	2.36	Cranial vena cava
P2	9.04	2.26	Auricular catheter
P3	8.85	2.21	Auricular catheter

**Table 4:** Intra-day relative bias (%).

Concentration Level (ng/mL)			Intra-Day Relative Bias (%)	
CBD	7-COOH-CBD	Series	CBD	7-COOH-CBD
0.5	5	1	0.5	3.0
0.5	5	2	−3.2	2.1
0.5	5	3	−7.4	2.6
1.5	15	1	−6.9	−6.4
1.5	15	2	−9.0	−2.7
1.5	15	3	3.6	−4.5
15	150	1	1.3	−0.8
15	150	2	−1.5	−1.4
15	150	3	4.9	−1.1
50	500	1	0.2	−3.5
50	500	2	0.2	−0.4
50	500	3	1.2	−2.0
**Concentration Level (ng/mL)**		**Inter-Day Relative Bias (%)**		
**CBD**	**7-COOH-CBD**	**CBD**	**7-COOH-CBD**	
0.5	5	−3.4	2.4	
1.5	1.5	−4.1	−3.0	
15	15	1.6	0.8	
50	500	0.5	−2.8	

**Table 5:** Repeatability and intermediate precision.

Concentration Level (ng/mL)		Repeatability (RSD%)		Intermediate Precision (RSD%)	
CBD	7-COOH-CBD	CBD	7-COOH-CBD	CBD	7-COOH-CBD
0.5	5	5.1	3.0	6.0	3.0
1.5	15	5.0	2.3	8.1	3.9
15	150	2.8	2.6	4.1	4.0
50	500	2.7	2.4	2.7	3.0

**Table 6:** Dilution integrity.

Replicate	CBD (20 ng/mL)	7-COOH-CBD (200 ng/mL)
1	−9.8	−4.7
2	−10.2	−5.1
3	−7.1	−5
4	−9.3	−1.6
5	−11.2	−5.2
Mean (±SD)	−9.5 ± 1.5	−4.3 ± 1.5

**Table 7:** Pharmacokinetic parameters depending on the used model and the number of subjects.

IV-Bolus Model	Two Comp.	Two Comp.	Three Comp.	Three Comp.
Number of Animals	*n* = 3	*n* = 2	*n* = 3	*n* = 2
PK Parameters	Values (±SD)			
A	542.53 ± 116.45	500.89 ± 129.30	640.14 ± 43.16	638.52 ± 60.91
α	3.71 ± 2.26	2.75 ± 2.18		12.28 ± 7.90
B	168.95 ± 95.92	133.91 ± 105.05		330.39 ± 41.34
β	0.34 ± 0.07	0.33 ± 0.10	0.70 ± 0.21	0.80 ± 0.19
C	-	-	10.56 ± 6.33	17.71 ± 15.74
γ	-	-	316.02 ± 38.40	0.09 ± 0.12
Statistics				
R^2^	0.988 ± 0.010	0.983 ± 0.10	0.997 ± 0.002	0.996 ± 0.002
AIC	−13.66 ± 5.21	−12.22 ± 6.47	−22.11 ± 8.22	−21.94 ± 11.62

**Table 8:** Fisher test value (*n* = 3); WRSS2 and WRSS3 are the weighted residual sum of the square of the two-compartment model and three-compartment model, respectively; DF2 = degrees of freedom of two-compartment model; DF3 = degrees of freedom of three-compartment model.

	Two-Compartment	Three-Compartment
Weighted residual sum of square (WRSS)	0.13 ± 0.07	0.04 ± 0.03
Degrees of freedom (DF)	6	4
$F=\frac{\;\frac{\left|WRSS2-WRSS3\right|}{\left|DF2-DF3\right|}}{\frac{WRSS3}{DF3}}=4.50$ F_Table for α = 0.05_ = 6.16		(1)

**Table 9:** PK parameters of CBD IV injected analyzed with a two-compartment model. V1 = volume of the central compartment; Vss = volume at steady state; k_1-0_, k_1-2_, k_2-1_ = micro constants; Cl = clearance; f_c_ = ratio of quantity in the central compartment over total quantity.

PK Parameters	Value (±SD)
V_1_ (mL)	1512.43 ± 538.41
V_ss_ (mL)	3260.35 ± 286.66
k_1-0_ (h^−1^)	1.04 ± 0.20
k_1-2_ (h^−1^)	1.80 ± 1.43
k_2-1_ (h^−1^)	1.20 ± 0.74
Depth (k_10_/k_12_)	0.58
Capacity (k_12_/k_21_)	1.50
Cl (mL·h^−1^)	1514.5 ± 261.16
f_c_	0.33

## Data Availability

The data presented in this work are available on demand.

## Supplementary Materials

The following supporting information can be downloaded at https://www.mdpi.com/article/10.3390/pharmaceutics16010140/s1: Table S1: Calibration standard preparation; Table S2: Validation standard preparation; Table S3: Quality control (QC) sample preparation; Table S4: Linearity; Figure S1: In vitro drug release of CBD within the CBD: HP-β-CD spray-dried complex in sink conditions; medium at pH 6.8 + 1% SLS; 37 °C, Withdrawals at 1, 2 and 5 min were filtered through PTFE 0.2 µm prior to HPLC analysis via validated method (*n* = 2).
