# Supplementary material for: An Intravenous Pharmacokinetic Study of Cannabidiol Solutions in Piglets through the Application of a Validated Ultra-High-Pressure Liquid Chromatography Coupled to Tandem Mass Spectrometry Method for the Simultaneous Quantification of CBD and Its Carboxylated Metabolite in Plasma
Source: Pharmaceutics. 2024 Jan 20;16(1):140. doi: 10.3390/pharmaceutics16010140 (PMC10820640; doi:10.3390/pharmaceutics16010140)
Supplement: Supplementary file 1 [file pharmaceutics-16-00140-s001.zip › pharmaceutics-2812331-supplementary.pdf]

Supplementary materials:

Table S1. Calibration standard preparation.

	Blank	Std 1	Std 2	Std 3	Std 4	Std 5	Std 6	
**CBD**	**0**	**0.50**	**2.0**	**5.0**	**10**	**25**	**50**	**ng/mL**
**7-COOH-CBD**	**0**	**5.0**	**20**	**50**	**100**	**250**	**500**	**ng/mL**
**Plasma**	500	450	480	450	480	450	450	μL
**SF1**	-	-	-	-	-	-	50	μL
**SF2**	-	-	-	-	20	50	-	μL
**SF3**	-	-	20	50	-	-	-	μL
**SF4**	-	50	-	-	-	-	-	μL

Table S2. Validation standard preparation.

	Val 1	Val 2	Val 3	Val 4	
**CBD**	**0.50**	**1.5**	**15**	**50**	**ng/mL**
**7-COOH-CBD**	**5.0**	**15**	**150**	**500**	**ng/mL**
**Plasma**	450	485	470	450	μL
**SF1**	-	-	-	50	μL
**SF2**	-	-	30	-	μL
**SF3**	-	15			μL
**SF4**	50	-	-	-	μL

Table S3. Quality control (QC) sample preparation.

	QC1	QC2	QC3	
**CBD**	**1.5**	**15**	**50**	**ng/mL**
**7-COOH-CBD**	**15**	**150**	**500**	**ng/mL**
**Plasma**	485	470	450	μL
**SF1**	-	-	50	μL
**SF2**	-	30	-	μL
**SF3**	15	-	-	μL

Table S4. Linearity.

	Linear regression model	r^2	r	Residual sum of square
CBD	y = 0.004757 + 1.006x	0.9987	0.9994	30.63
7-COOH-CBD	y = 1.505 + 0.9717 x	0.9985	0.9992	3509

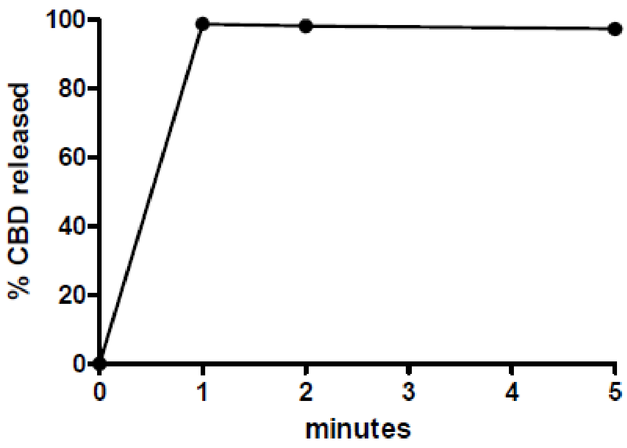

Figure S1. *In vitro* drug release of CBD within the CBD: HP-β-CD spray-dried complex in sink conditions; medium at pH 6.8 + 1% SLS; 37 °C, Withdrawals at 1, 2 and 5 minutes were filtered through PTFE 0.2 μm prior to HPLC analysis via validated method (n = 2).
